# Respiratory hospitalizations and wildfire smoke: a spatiotemporal analysis of an extreme firestorm in San Diego County, California

**DOI:** 10.1097/EE9.0000000000000114

**Published:** 2020-10-01

**Authors:** Rosana Aguilera, Kristen Hansen, Alexander Gershunov, Sindana D. Ilango, Paige Sheridan, Tarik Benmarhnia

**Affiliations:** a Scripps Institution of Oceanography, University of California San Diego; b Department of Family Medicine and Public Health, University of California San Diego, La Jolla; and; c School of Public Health, San Diego State University, San Diego, California

**Keywords:** California, Respiratory health, Spatiotemporal, Wildfire, California, Respiratory health, Spatiotemporal, Wildfire

## Abstract

Wildfire smoke adversely impacts respiratory health as fine particles can penetrate deeply into the lungs. Epidemiological studies of differential impacts typically target population subgroups in terms of vulnerability to wildfire smoke. Such information is useful to customize smoke warnings and mitigation actions for specific groups of individuals. In addition to individual vulnerability, it is also important to assess spatial patterns of health impacts to identify vulnerable communities and tailor public health actions during wildfire smoke events.

**Methods:**

We assess the spatiotemporal variation in respiratory hospitalizations in San Diego County during a set of major wildfires in 2007, which led to a substantial public health burden. We propose a spatial within-community matched design analysis, adapted to the study of wildfire impacts, coupled with a Bayesian Hierarchical Model, that explicitly considers the spatial variation of respiratory health associated with smoke exposure, compared to reference periods before and after wildfires. We estimate the signal-to-noise ratio to ultimately gauge the precision of the Bayesian model output.

**Results:**

We find the highest excess hospitalizations in areas covered by smoke, mainly ZIP codes contained by and immediately downwind of wildfire perimeters, and that excess hospitalizations tend to follow the distribution of smoke plumes across space (ZIP codes) and time (days).

**Conclusions:**

Analyzing the spatiotemporal evolution of exposure to wildfire smoke is necessary due to variations in smoke plume extent, particularly in this region where the most damaging wildfires are associated with strong wind conditions.

## Abstract 2

Supplemental Digital Content is available in the text.

**What this study adds**

Our spatiotemporal approach to investigate patterns of respiratory health impacts associated with exposure to wildfire smoke clearly showed that the highest excess hospitalizations were concentrated in areas downwind of wildfires. The temporal evolution of excess hospitalizations was also evident and mainly driven by wind patterns that first transported smoke offshore and later in the opposite direction. To our knowledge, our analyses are the first to investigate the variation respiratory health impacts considering both fine spatial and temporal dimensions, a particularly necessary endeavor in regions where wildfires are associated with strong winds that contribute to ignitions, spread flames, and transport smoke particles, thus increasing population exposure.

## Introduction

Wildfire smoke adversely impacts public health, notably through respiratory diseases,^1–3^ as fine particulate matter can penetrate deeply into the lungs.^4^ Several literature reviews have characterized how fine particles from wildfire smoke, an acute exposure and episodic in nature, have influenced morbidity and mortality across many regions.^2,5–8^ Wildfire fine particulate matter in the US is projected to increase with global change,^9^ as well as the associated health impacts of exposure to wildfire smoke.^10,11^

Despite recent efforts in fire and fuel management to prevent large wildfires from occurring,^12,13^ wildfire smoke continues to reach coastal zones and impact large populations. In this context, early warning systems, including advisories combined with emergency public health measures, have been developed to prevent the burden associated with exposure to wildfire smoke.^14^ Epidemiological studies of differential impacts of wildfire smoke exposure have mainly targeted population subgroups (such as children, the elderly or individuals with preexisting respiratory conditions such as asthma) that may be more vulnerable to the effects of wildfire smoke.^15–18^ Such information can be particularly useful to target specific individuals when smoke warning systems are activated. In addition to individual vulnerability, it is also important to assess the spatial variability of wildfire smoke health impacts to identify vulnerable communities for tailoring public health actions during wildfire smoke events.

Population exposure levels to wildfire smoke vary widely depending on population location, the area burned, fuels, fire intensity, rate of spread, and meteorological factors such as wind.^6,8^ In Southern California (SoCal), the largest fires are typically driven by the onset of desiccating Santa Ana winds (SAWs) in early fall and before the arrival of winter precipitation, which in turn can promote further fuel drying.^19^ SAW-driven wildfires burn in the backcountry foothills and transport smoke towards coastal areas, where most of the population is concentrated. As the offshore (easterly) SAWs subside, the return of prevailing onshore winds can spread smoke particles over a broader coastal and inland area. Fall wildfires in this region tend to last longer than wildfires at other times of the year^20^ and cause the greatest damage to human health and property.^21,22^ Furthermore, SoCal is estimated to experience the highest percentage increase in respiratory admissions from wildfire smoke in coastal Western US.^10^

In this study, we aimed to assess the spatial variation in respiratory hospital admissions in San Diego County during a set of major wildfires in October 2007, which led to a substantial public health burden.^17^ For this purpose, we propose a within-community matched design^23^ adapted to assess the differential effects on respiratory health due to smoke exposure, compared to reference periods before and after wildfires, while explicitly accounting for the spatial variation of such effects. We conducted sensitivity analyses using the 2007 wildfires as well as similar wildfires that took place in October 2003, in terms of magnitude and location. In addition, we conducted a falsification test by applying our approach for the same dates in two years without wildfire activity (2000 and 2004).

To our knowledge, no study has previously conducted a spatiotemporal analysis of health impacts associated with exposure to wildfire smoke. Unlike previous efforts that typically report global estimates for a region and/or wildfire event, we identify and quantify excess hospitalizations related to wildfire smoke pollution resolving finer spatial (ZIP code) and temporal (daily) scales. Analyzing exposure both across space and time is warranted due to variations in smoke plume extent and associated exposure to wildfire air pollution.

## Methods

### Study setting

**Figure 1.**
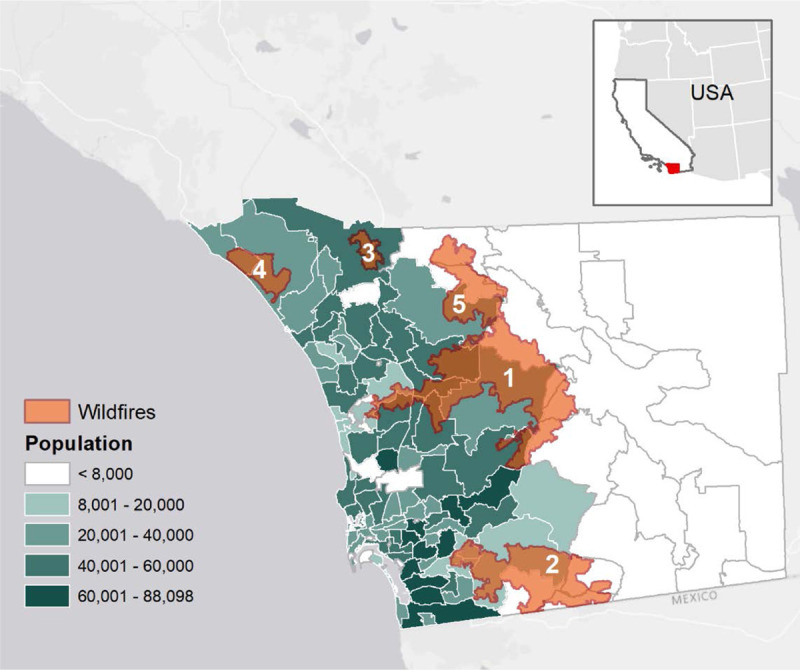
Population in each of the 108 ZIP codes in San Diego County, California, USA (location is shown in red; inset figure). Wildfire total perimeters for October 2007 are also shown: 1 – Witch and 2 – Harris ignited on October 21, 3 – Rice on the 22nd, 4-Ammo and 5-Poomacha on the 23rd (Table 1). Santa Ana winds, blowing from the North East, can transport smoke from wildfires burning inland to the most populated coastal areas.

More than two dozen major fires broke out between October 20 and 23, driven by strong SAWs and burning over 972,000 acres across SoCal. Overall, the 2007 SoCal wildfires imposed $3.4 million in health care costs for hospital and emergency department visits alone.^24^ Three of the largest fires (Witch, Harris, and Poomacha, Figure 1, Table 1) burned on the western slopes of the coastal ranges in San Diego County with smoke blown towards the densely populated coastal zone. Hutchinson et al.^17^ found that respiratory diagnoses, especially asthma, were elevated during these wildfires for the beneficiaries of a low-cost health coverage program targeting children and adults with low income and resources.

**Table 1.** Characteristics of the five major wildfires that burned in San Diego County during October 2007

Number in Figure 1	Wildfire name	Ignition date	Total area (acres)
1	Witch	October 21, 2007	197,000
2	Harris	October 21, 2007	90,728
3	Rice	October 22, 2007	9,472
4	Ammo	October 23, 2007	21,084
5	Poomacha	October 23, 2007	49,390

SoCal experienced a similar set of wildfires in October 2003, fanned by moderate SAWs,^19^ and affecting human health.^1^ In San Diego County, the largest fire (Cedar) started on October 25, burning almost 300,000 acres. The severity of the immediate human impact of the October 2003 and 2007 wildfires was exacerbated by the rapid growth of an extensive Wildland-Urban Interface (WUI) proximate to a large population in SoCal.^19,25^ Due to similarities in fire areas burned, fire ignition dates, and both October 2003 and 2007 firestorms being SAW-driven, we used the 2003 events to test the effectiveness of our within-community matched design in quantifying excess counts for hospitalizations and their spatiotemporal variation.

### Exposure to wildfire smoke

The study area covered 108 ZIP code polygons in San Diego county (Figure 1). Fire perimeters were obtained from the Fire and Resource Assessment Program (http://frap.fire.ca.gov/) of the California Department of Forestry and Fire Protection. The fire perimeters were used to identify ZIP codes directly affected by the burned area of each wildfire (i.e., ZIP codes overlapping with fire perimeters). To assess the extent and exposure to wildfire smoke plumes, we visually described the temporal evolution and assessed the spatial extent of the smoke plumes using satellite imagery from the Moderate Resolution Imaging Spectroradiometer (MODIS) Rapid Response System (https://modis.gsfc.nasa.gov/). By combining the information provided by fire perimeters and satellite imagery showing smoke plumes, we were able to determine which days and ZIP codes were exposed to wildfire smoke for both 2007 and 2003 firestorms.

In addition, smoke plumes obtained from the NOAA Hazard Mapping System (HMS; https://satepsanone.nesdis.noaa.gov/pub/volcano/FIRE/HMS_ARCHIVE/) were available for the 2007 firestorm (eFigure 2; http://links.lww.com/EE/A107). The HMS product uses visible satellite imagery and trained satellite analyst skills to estimate the spatial extent of smoke, though it cannot discern whether a given plume is at ground level or higher in the atmosphere.

### Respiratory health data

We used daily clinical data for hospitalizations for respiratory diseases from the Office of Statewide Health Planning and Development database of patient discharges for the study period and all ZIP codes in San Diego County. Respiratory hospitalizations correspond to pulmonary diagnoses such as asthma, chronic obstructive pulmonary disease, pneumonia, and interstitial lung disease-9 (codes 460–519). Number of hospitalizations was then aggregated at the daily level by ZIP code. ZIP codes reported in the database correspond to the patient’s residential address. The total population based on the 2010 US Census for San Diego County was 3.1 million.

### Within-community matched design: a spatiotemporal approach

We propose a spatial within-community matched design,^23^ adapted to the examination of wildfire impacts, to analyze the spatial variation of ZIP code level hospital admissions attributable to the 2007 wildfire smoke. Each ZIP code’s hospital admission daily rate within a 5-day exposed period was compared to ZIP code-specific control periods that are matched to the same day of the week within a 2-week period before and after the given exposed day. This design has the advantage of controlling for any (measured or not) time-fixed ZIP code-level confounding factors.

Exposed days (October 22–26) were selected based on previous evidence of the 2007 wildfires and their impacts on public health.^17^ We further assessed this period by the presence of SAWs and smoke plumes (based on satellite imagery) starting October 21–22 and considering that the areal growth of the fires lessened by October 25–26, when SAWs stopped and the prevailing onshore winds returned.^26^ Bidirectional control days were selected on the same day of the week as the exposed days, during the 2 weeks before (October 8–21) and after (October 27–November 9).

We then compared, for each ZIP code, the number of admissions between exposed and control days. We first identify four control days (i.e., baseline) for each exposed day taking the same week day on the previous two and next two weeks in the same ZIP code. We use the median of these 4 control days as the contrast for each exposed day. We subtract this median from the exposed day admissions count to form our difference. For plotting purposes, we average over the 5 exposed day contrasts. We thus estimated the excess count of admissions for a given exposed day and compared the spatial distribution of any pattern, or lack thereof, to the extent of the smoke plumes across the study region. For this purpose, we used the geospatial datasets (see Exposure to wildfire smoke).

In addition to our primary analysis, we conducted a sensitivity analysis by looking at different exposed and corresponding control days for the same 2007 wildfire events, as well as similar wildfires in October 2003 in terms of magnitude and location. Lastly, we conducted a falsification test by applying our approach above to the same time period but in 2 years without wildfire activity (October 2000 and 2004) in the region. These two periods were selected based on the absence of wildfire in October and the preceding month. Falsification hypotheses can help adjudicate whether observational associations are robust. We expected a lack of specific spatiotemporal patterns in respiratory hospitalizations in the absence of wildfires.

Spatial statistical tools were implemented after the analyses described above, to examine spatial clustering. A local indicator of spatial autocorrelation, Anselin Local Moran’s I was used to examine spatial clusters of ZIP codes with counts of excess hospitalizations in years with wildfires (2007 and 2003) when we expect an aggregation of high values nearby fire perimeters. We used the Moran’s I Global index of spatial autocorrelation to assess the similarity, or spatial dependence, across zip codes with respect to excess counts of respiratory hospitalizations in years without wildfires (2000 and 2004).

### Bayesian Hierarchical Model

We use a spatial Bayesian Hierarchical Model (BHM) to assess and increase precision in our estimates for the 2007 firestorm outcomes. BHM provides a decrease in variance of estimations by leveraging observations in nearby locations to provide estimates in any given ZIP code. BHM reduces variation in such estimates due to aggregation, thus increasing precision. We use all 5 exposed day outcomes aggregated (i.e., differences in hospitalization counts in all 5 exposed days added together) from our 2007 within-community matched design analysis as the response value in a linear BHM, using the *spBayes* package in R.^27^ Because point-referenced data are required for BHMs, we used population-weighted centroids provided by the US Census Bureau as location for our ZIP codes.

We fit an empirical semivariogram to estimate the starting prior to the parameters: sill (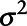), nugget (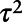), and range (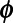). We used a spherical covariance structure based on the shape of the empirical semivariogram. The hierarchical model involves the following two stages:


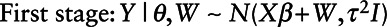



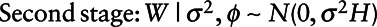


where *W* is the vector of spatial random effects and 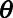 is the vector of parameters including 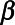, sill, nugget, and range. The *Y*_i_ are conditionally independent given *W*. *H* is the spatial correlation structure, in this case, we specify it as spherical and isotropic. The second stage model is the process model introduced to capture spatial dependence. We completed model specification by adding priors to 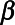 and 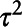, and the hyperparameters 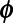 and 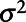. This all can be viewed as a Bayesian spatial extension to a general linear model. Our *X* is only using an intercept and therefore the full model captures the spatial process underlying the distribution of excess hospitalizations in San Diego county during the 5 exposed days.

We used prior distributions of parameters such that the model is not particularly sensitive to the priors we input. We used 10,000 Markov chain Monte Carlo samples, 7,500 of which were used for burn in. Recovered samples of spatial weights were used for the estimation and interpolation across space using multi-level B-splines on a 300 × 300 raster grid. Though the above methodology assumes isotropy, we acknowledge that spatial correlation across grid cells in our study area will differ based on climatic and topographic characteristics.

Lastly, to represent the precision of the BHM estimates, we estimate the signal-to-noise ratio (SNR) using the resulting model output (weights for each ZIP code and standard deviations). To estimate the SNR, we take the excess count estimates for each ZIP code and divide it by the standard deviation by ZIP code provided from the BHM output. The SNR is thus mapped for each ZIP code as an indication of areas where estimates of excess hospitalizations are more (or less) precise. Traditionally, precision is found at an SNR of about 2, due to the scarcity of the data for this study SNR as high as 2 was not expected.

## Results

### Characteristics of wildfires, smoke exposure, and respiratory hospitalizations

The wildfires were continuously fanned by the SAWs, increasing the burned area and delineating clear smoke pathways directed to coastal ZIP codes (e.g., eFigures 1, 2, and 4C; http://links.lww.com/EE/A107). Once these offshore winds weakened around October 25, smoke was widespread across the entire county (eFigure 1; http://links.lww.com/EE/A107). By October 26, onshore winds started blowing smoke particulate matter back towards inland ZIP codes (eFigure 2; http://links.lww.com/EE/A107). In addition, the progression of fire perimeters (eFigure 3; http://links.lww.com/EE/A107) showed that by this day most of the wildfires in the county had reached the total area attributed to the final perimeters as shown in Figure 1.

Monthly mean values for counts of hospitalizations for respiratory conditions in the entire County were similar during the fall months of 2000, 2003, 2004, and 2007 (Table 2). The highest maximum number of hospitalization counts (8) in a given ZIP code, as well as for total counts in the study region, was however observed in October 2007. Considering the spatial distribution of hospitalization counts for October 22, 2007 (eFigure 3; http://links.lww.com/EE/A107), these higher counts were concentrated in ZIP codes located immediately downwind of the largest wildfire whereas this pattern, or any other, was not observed on the same day in a nonwildfire period, for example, 2004.

**Table 2.** Hospital admissions for respiratory conditions during the months of fall (September, October, and November) during wildfires (2003 and 2007) and during periods without wildfire in 2000 and 2004

Hospital Admissions for Respiratory Conditions in San Diego County						
With wildfire						
Descriptive statistic (based on daily values)	2003			2007		
	September	October	November	September	October	November
Mean	0.37	0.41	0.46	0.36	0.44	0.39
SD	0.70	0.79	0.81	0.71	0.81	0.71
Max	5	8	6	6	8	5
Sum	1,199	1,372	1,482	1,163	1,483	1,274
Without wildfire						
Descriptive statistic (based on daily values)	2000			2004		
	September	October	November	September	October	November
Mean	0.41	0.44	0.51	0.32	0.35	0.42
SD	0.77	0.81	0.89	0.62	0.68	0.77
Max	6	6	6	4	5	5
Sum	1,315	1,489	1,642	1,037	1,164	1,347

Global monthly values are based on daily hospitalizations throughout the 108 ZIP codes pertaining to San Diego County.

### Spatiotemporal patterns of excess hospitalizations

**Figure 2.**
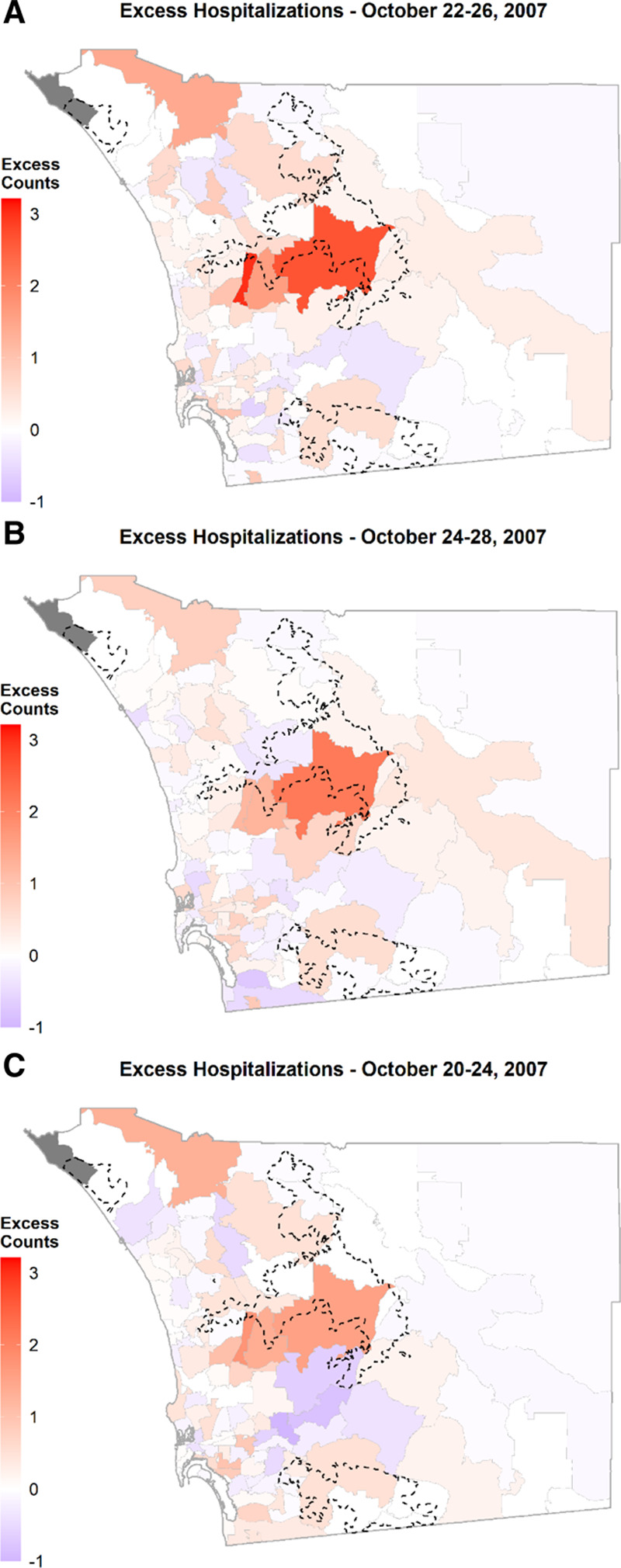
Mean excess counts for admissions during the 2007 selected exposed days. A, The main exposed 5-day period, October 22–26, is compared to a set of two different 5-day periods (B, C). The 5-day period between October 22–26 yielded the highest mean excess count of admissions, concentrated mainly in the surroundings, and particularly downwind with respect to SAW, of the Witch Fire. Fire perimeters in (A–C) correspond to the total area burned.

**Figure 3.**
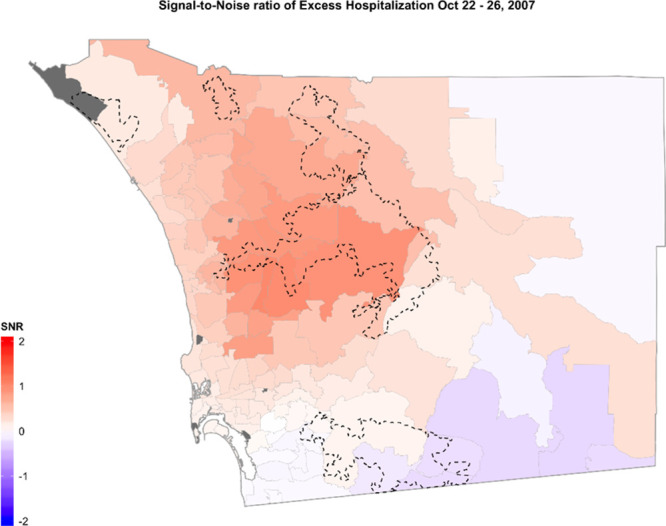
Signal-to-noise ratio (SNR) per ZIP code, based on estimates of the Hierarchical Bayesian Model of the October 22–26, 2007 exposed days. Higher SNR values indicate higher precision in our estimates and are concentrated in ZIP codes covered by and downwind of the Witch fire.

Our spatiotemporal approach revealed an aggregation of admissions for respiratory conditions, particularly those ZIP codes where wildfires were burning and on ZIP codes located to the west of the fire perimeters. Based on the Local Moran’s I index, clustering of high values of excess counts were mainly found downwind of the Witch Fire (eFigure 5; http://links.lww.com/EE/A107). For the exposed period of October 22–26, excess counts represented 30% of total counts of admissions for respiratory conditions in the county (Table 3). The mean number of excess admissions during the exposed 5-day period was 3. The ZIP code with the largest excess count of admissions overlapped with the Witch Fire perimeter (Figure 2A). In the exposed of our BHM, this same area showed the highest mean counts in excess hospitalizations (eFigure 4; http://links.lww.com/EE/A107) and the highest precision for the BHM estimates (Figure 3).

**Table 3.** Excess respiratory hospitalizations on each exposed day as compared with control days, that is, same day of the week 2 weeks before and after the exposed period

Wildfire period – exposed days	Excess counts of respiratory hospitalizations					
	October 2007			October 2003		
	20–24	22–26(*)	24–28	22–26	24–28	26–30(*)
Day 1	–6	28	19	7	–16	6
Day 2	1	13	27	–10	–2	12
Day 3	28	19	10	–16	6	–1
Day 4	13	27	7	–2	12	5
Day 5	19	10	–5	6	–1	8
Total excess	54	96	58	–14	–1	30
Total counts	273	325	267	199	221	257

Exposed 5-day periods include three sets during the October 2003 and 2007 wildfires, with associated exposure to smoke. This table also displays the total respiratory hospitalizations for each exposed 5-day period in San Diego County. (*) indicates the main exposed 5-day period for each year.

Total excess counts for each of the additional two sets of exposed periods represented 20% of the total admissions. As shown in Figures 2B, C, the mean excess count per ZIP code was also lower than in our main period above, confirming that the greatest exposure and impact on health was registered immediately after ignition of the largest fires on October 21 (i.e., exposed days October 22–26).

**Figure 4.**
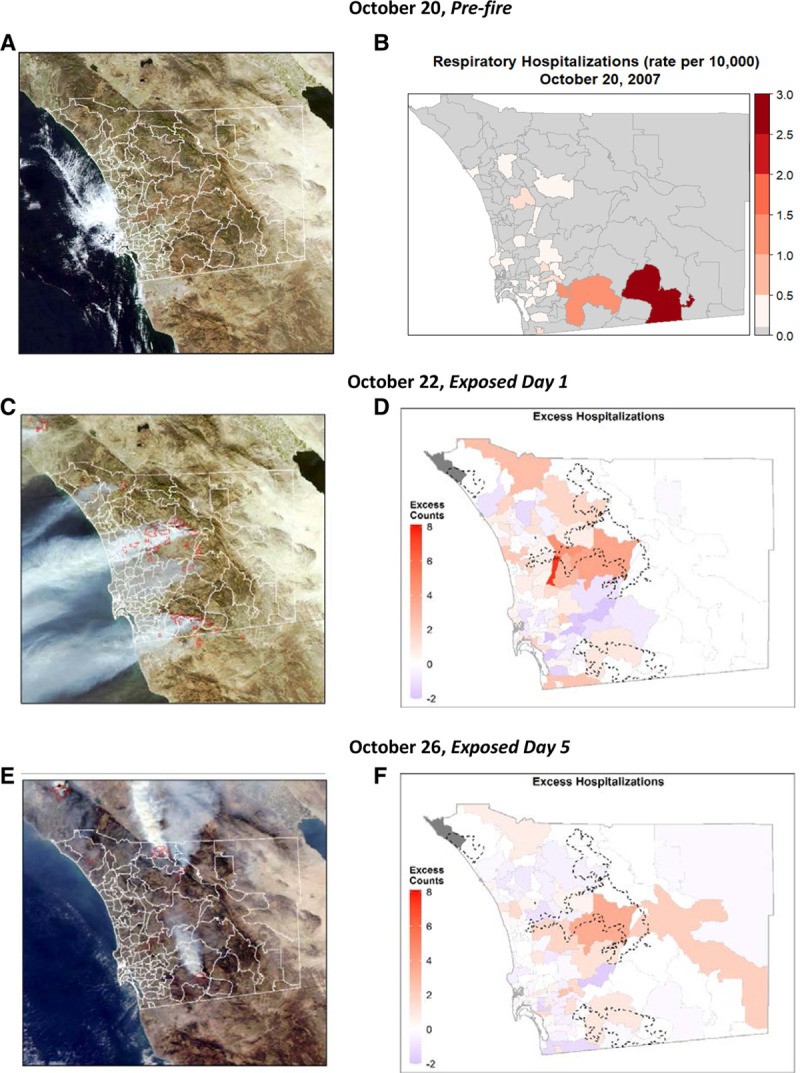
Prefire conditions were observed on October 20, 2007, in terms of (lack of) visible smoke (A) and spatial distribution of hospitalizations for respiratory conditions (B). The extent of the smoke plume and the spatiotemporal evolution of excess counts of admissions are shown for exposed days 1 (October 22; C, D) and 5 (October 26; E, F). Red perimeters in satellite imagery (C, E) show the location of thermal anomalies, identified as fires, detected by MODIS using data from middle infrared and thermal infrared bands.

For the selected exposed period of October 22–26, MODIS images provided a visual assessment of wildfire smoke dispersion and subsequent exposure, and showed that, compared to the pre-fire conditions when the air was clear (Figure 4A), smoke from the wildfires burning in the backcountry reached coastal areas and the Pacific Ocean (Figure 4C) by the action of strong SAWs. For reference, prefire rates of hospitalizations are shown in Figure 4B. On October 22, the day after the largest fires had ignited, the excess count for respiratory conditions in a given ZIP code reached a high of 6 (Figure 4D). Once these offshore winds weakened, around October 25, the smoke plume drifted towards inland areas with the onset of onshore winds. The next day, we observed migration of increased excess counts towards inland ZIP codes, following smoke dispersed back inland (Figure 4F).

**Figure 5.**
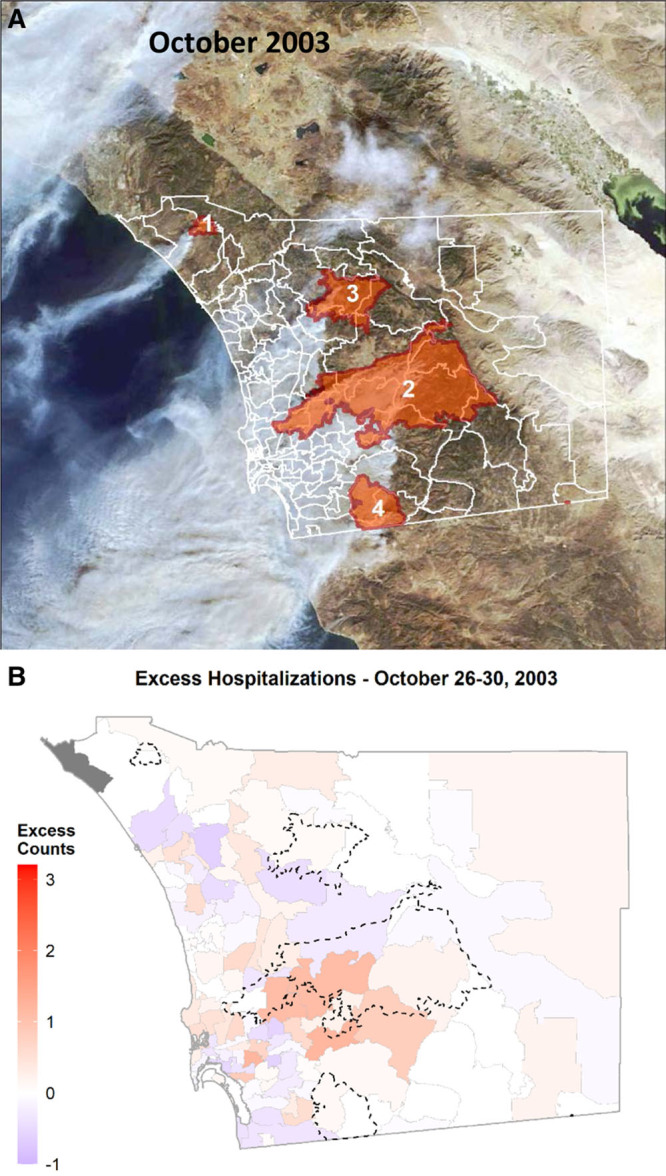
A, Fire total perimeters for October 2003 (1 – Roblar 2, 6,900 acres; 2 – Cedar, 270,000 acres; 3 – Paradise, 57,000 acres; 4 – Mine/Otay, 45,000 acres). The MODIS satellite image shows the smoke plumes on October 26, 2003. B, Mean excess counts for admissions during the 2003 main exposed days (A) October 26–30, compared to a set of two different exposed 5-day periods. The 5-day period between October 26–30 yielded the highest mean excess count of admissions, concentrated mainly and immediately downwind of the Cedar Fire, which started burning on October 25.

**Figure 6.**
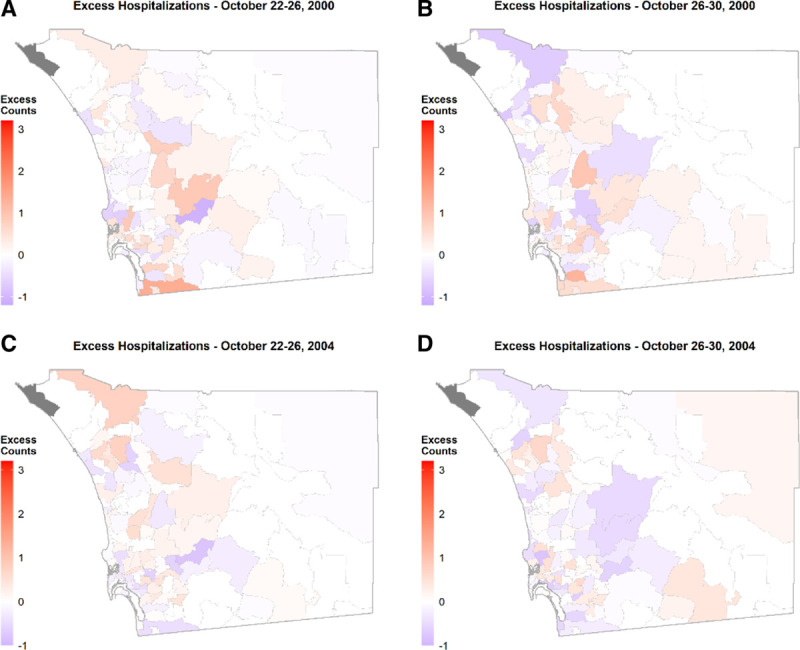
During the falsification periods in October 2000 and 2004, no specific spatial pattern was observed in excess counts of admissions in the county. Spatial autocorrelation was assessed via. Moran’s I (see eTable 1; http://links.lww.com/EE/A107 for test statistics). In all cases, spatial patterns were found to be random. In addition, these values were considerably lower than those estimated for the same period(s) in October 2007.

The two other case-day periods for 2007 yielded lower counts of excess hospitalizations overall and at the individual ZIP code level (Table 3; Figure 2B, C). The case-day period with the highest excess hospitalizations for the October 2003 wildfires was between October 26–30 and represented 12% of county total. This period experienced widespread smoke, particularly downwind of the wildfires (e.g., Figure 5A). Figure 5B shows that the highest excess counts were found immediately downwind of the Cedar Fire, which was also confirmed by Local Moran’s I test, as shown in eFigure 5; http://links.lww.com/EE/A107. In terms of October 2000 and 2004, the falsification test periods without wildfire smoke, a noisy pattern in space and time was observed (Figure 6). This absence of specific patterns was further confirmed by a set of Global Moran’s I tests presented in eTable 1; http://links.lww.com/EE/A107. When comparing individual exposed days for the 22–26 period, the excess counts in a given exposed day in 2007 were at least 9–28 counts higher than during 2004 but not necessarily in 2000 (Table 4). The excess counts for this period in 2000 and 2004 represented 8 and 5% of the total counts for the respective respiratory hospitalizations in the county.

**Table 4.** Excess respiratory hospitalizations on each exposed day as compared to control days, that is, same day of the week 2 weeks before and after the exposed period

Excess counts of respiratory hospitalizations				
Without wildfire – exposed days	October 2000		October 2004	
	22–26	26–30	22–26	26–30
Day 1	–2	22	0	–1
Day 2	–8	2	3	4
Day 3	0	–4	3	–1
Day 4	10	6	5	–10
Day 5	22	–4	–1	–8
Total excess	23	23	10	–15
Total count	274	261	198	187

Exposed 5-day periods include two periods without wildfires in October 2000 and 2004, which correspond to the main exposed periods in October 2007 (22–26) and 2003 (26–30). This table also displays the total respiratory hospitalizations for each exposed 5-day period in San Diego County.

## Discussion

In this study, we found that individuals in ZIP codes exposed to wildfire smoke were the most affected in terms of increased number of excess hospitalizations during the October 2007 firestorm, with respect to control days prefire and postfire. In addition, the three largest wildfires (Witch, Harris, and Poomacha) in October 2007 burned from 17 to 27 % of areas that had burned in the past 5 years.^20^ Specifically, there was extensive overlap between these fires and those that occurred in San Diego County during the October 2003 firestorm. Therefore, because most destructive wildfires in the region tend to break out in the backcountry slopes where SAWs are the strongest, wildfire-prone areas can be identified, both historically (e.g., Wildfire Hazard Potential by the USDA Forest Service) and in specific weather forecasts and warnings (e.g., National Weather Service Red Flag Warnings).

In spite of warnings and evacuation notifications issued soon after the start of the fires in 2007, which prompted an unprecedented large-scale evacuation in the county, only 42% of the households in the affected areas received a notification through a computerized mass-communication system.^28^ Though many other channels of communication, such as the media, police, and fire rescue sirens and loudspeakers and responders going door-to-door, were used to notify communities at risk, individuals in affected areas could have failed to timely evacuate the area and thus extend their exposure to thick smoke in the immediate proximity to the fires. In addition, cultural and linguistic obstacles were encountered when addressing the largely diverse population in the county,^28^ and some communities in the proximity of the fires might have not been fully aware of the evacuation notifications.

Based on the smoke transport driven by the offshore SAWs, we also expected a significant increase of excess hospitalizations in most of the vastly populated coastal ZIP codes covered by the plumes. Hutchinson et al.,^17^ modeled the fine particulate matter (PM_2.5_) levels during these 2007 wildfire events and found that most of the western part of the county greatly exceeded the US EPA 24-hour air quality standard of 35 μg/m^3^. Though coastal ZIP codes were also shown to be affected by increases in PM_2.5_, as smoke moves downwind, it can become more diluted and often more widespread, as seen on satellite images (e.g., Figure 3C). Furthermore, with wildfires burning in inland areas, it is not unexpected that the surrounding ZIP codes and those immediately downwind show the greatest impacts in terms of excess hospitalizations for respiratory conditions.

Emission factors, which specify the mass of a pollutant emitted per unit mass of biomass burned, for fine particles in smoke are highly dependent on fuel characteristics, stage of combustion, and burn conditions.^5^ Although public health regulatory decisions are more relevant to the potency values (i.e., PM_2.5_ mass), the emission factors reflect real-world exposures and can provide further insight while assessing the health effects of wildland fires.^29^ This information is; however, often difficult to obtain and approaches like our spatial within-community matched design can help identify the differential effects, in time and space, of wildfire exposure with respect to fire and smoke characteristics.

Study limitations include the use of patient home address to estimate exposures, though this is an intrinsic characteristic of our dataset as is the case in most epidemiological studies. Though we considered hospitalizations at the daily level, within-day heterogeneity in health outcomes^30^ may also be relevant for future spatiotemporal studies that consider finer scales in the study of wildfire impacts. Overall, the within-community matched design analysis presented in our article, together with the examination of smoke plumes using satellite imagery, smoke plume polygons, and previous publications that studied the 2007 and 2003 wildfires in great detail, we aimed to provide an alternative to modeling pollution for exposure taking advantage of the wind-driven nature of wildfires in the region. In addition, our approach can be extended to different exposures as well as different temporal and spatial scales.

Most studies addressing differential respiratory effects from wildfire smoke exposure tend to make this distinction based on characteristics of certain individuals, such as young children, those with preexisting cardiopulmonary conditions, and smokers. Identifying specific vulnerabilities to wildfire smoke helps targeting these population groups for preventive measures and health monitoring. In spite of the obvious threat that communities in the proximity of a wildfire can experience, the examination of spatiotemporal patterns in the response of adverse health outcomes has not received much attention in the literature.

Understanding the spatial variation of impacts of wildfire on public health is of vital importance and particularly relevant in a wildfire-prone region like SoCal that is densely populated downwind of wildfire-risk areas. In addition to an expected increase in wildfire frequency and severity in a warming climate,^31^ as well as a potential gradual shift of the wildfire season from fall to winter,^32^ the region is continuously experiencing spatial patterns of population expansion from coastal areas, leading to the expansion of the WUI,^19^ potentially increasing sources of wildfire ignition. All the above conditions lead to an increased probability of large and destructive wildfires during SAW events with downwind impacts on respiratory health.

## Conflicts of interest statement

The authors declare that they have no conflicts of interest with regard to the content of this report.

## Acknowledgements

This work was funded by the University of California Office of the President via. Multicampus Research Programs and Initiatives (MRPI; Climate and Health Interdisciplinary Research Program, MRP-17-446315) and the National Oceanic and Atmospheric Administration’s Regional Integrated Sciences and Assessments (RISA) California–Nevada Climate Applications Program award NA17OAR4310284.

## Supplementary Material


