# Supplementary material for: Respiratory hospitalizations and wildfire smoke: a spatiotemporal analysis of an extreme firestorm in San Diego County, California
Source: Environ Epidemiol. 2020 Oct 1;4(5):e114. doi: 10.1097/EE9.0000000000000114 (PMC7941788; doi:10.1097/EE9.0000000000000114)
Supplement: Supplementary file 1 [file ee9-4-e114-s001.docx]

**Supplemental Material**

**Respiratory Hospitalizations and Wildfire Smoke: A spatio-temporal analysis of an extreme firestorm in San Diego County, California**

Rosana Aguilera^1§^, Kristen Hansen^2§^, Alexander Gershunov^1^, Sindana D. Ilango^2,3^, Paige Sheridan^2,3^, Tarik Benmarhnia^1,2^

^(1)^ Scripps Institution of Oceanography, University of California San Diego, La Jolla, CA, USA

^(2)^ Department of Family Medicine and Public Health, University of California San Diego, La Jolla, CA, USA

^(3)^ School of Public Health, San Diego State University, San Diego, CA

^§^ Equal contribution

Address correspondence to R. Aguilera, Scripps Institution of Oceanography, 9500 Gilman Drive #0230, La Jolla, CA, 92093, USA. Telephone: (805) 280-6219

E-mail: [r1aguilerabecker@ucsd.edu](mailto:r1aguilerabecker@ucsd.edu)

Figure S1: Additional MODIS satellite images for 2007 showing (a) the onset of Santa Ana winds (SAWs) and the start of the Harris Fires on October 21, (b) the smoke plumes reaching coastal areas by October 23, (c) the weakening of SAWs and the associated further dispersion of smoke towards inland areas, and (d) the widespread of smoke cause by the absence of SAWs and the onset of the typical onshore winds.

| 1. October 21 | 1. October 23 |
| --- | --- |
| 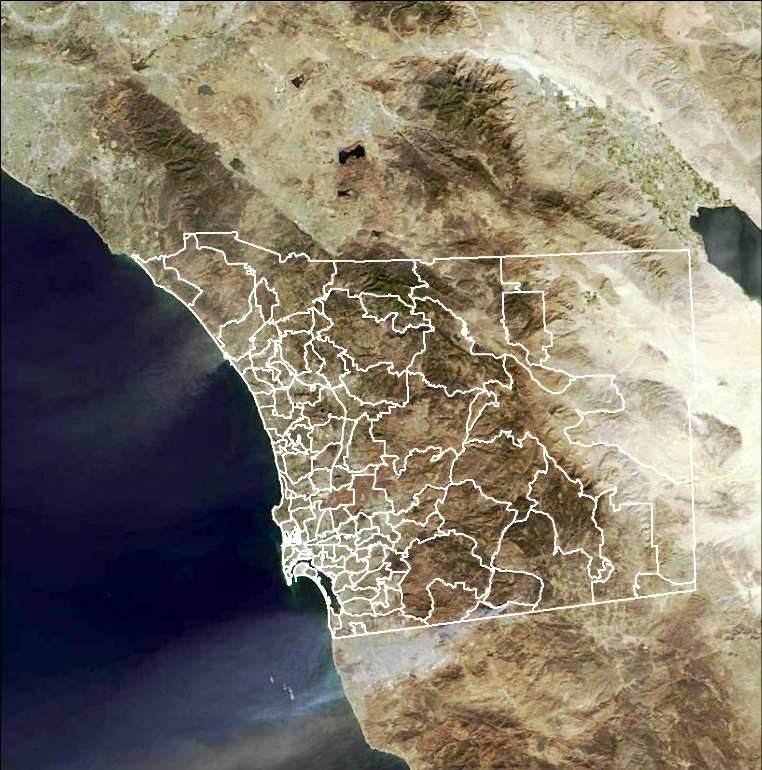 | 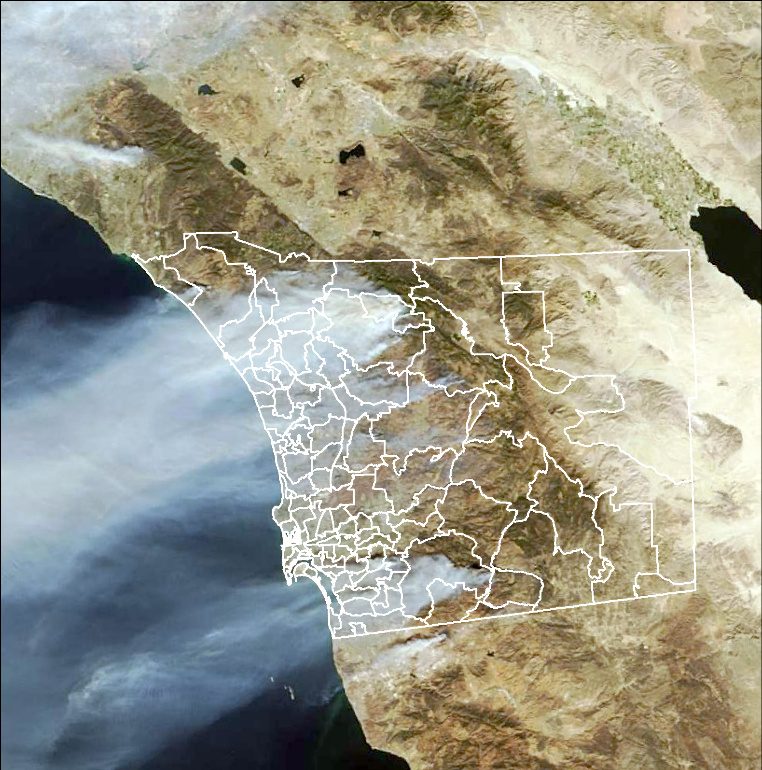 |
| 1. October 24 | 1. October 25 |
| 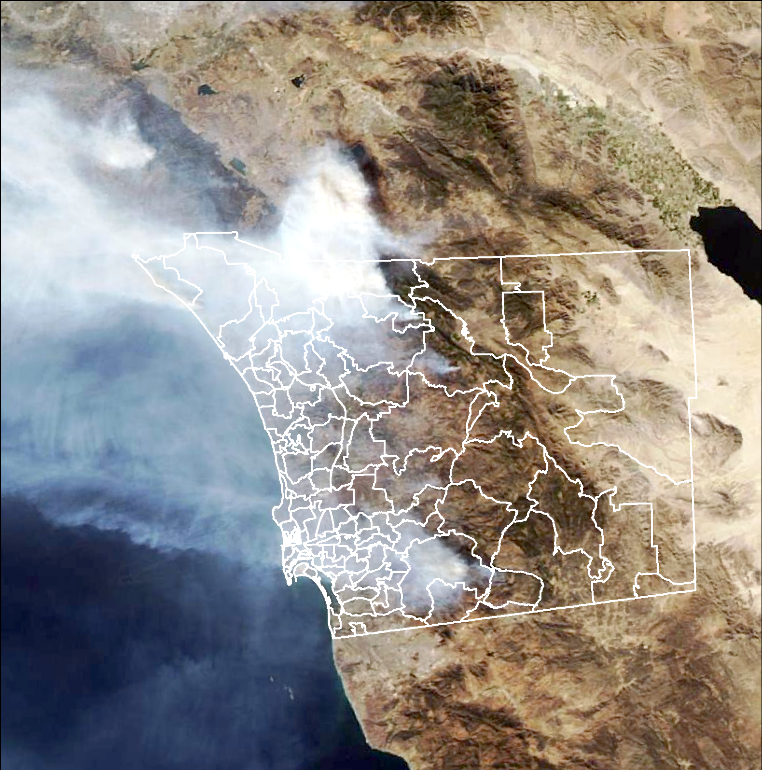 | 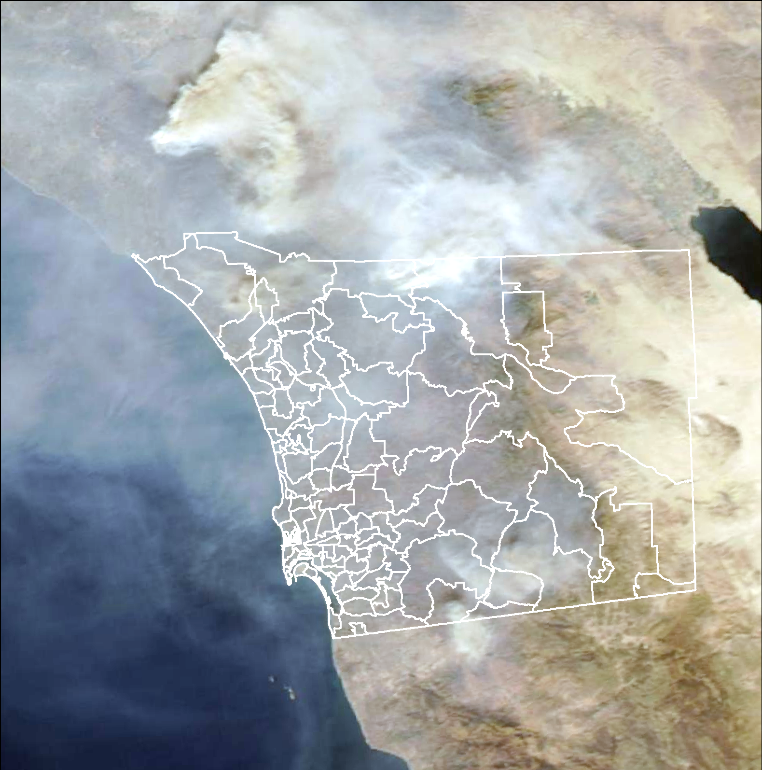 |

Figure S2: Top: Smoke plume extent for October 22 (left) and October 26, 2007 (right). NOAA Hazard Mapping System (HMS) smoke products represent the spatial extent of daily smoke plumes. Bottom: Progression of the wildfire perimeters during the 5-day exposed period in October 2007, starting October 22^nd^. By the end of such period (October 26), most wildfires had reached their final fire perimeters. (*) Ammo Fire evolving perimeters were only available for October 22-26, 2007.

| 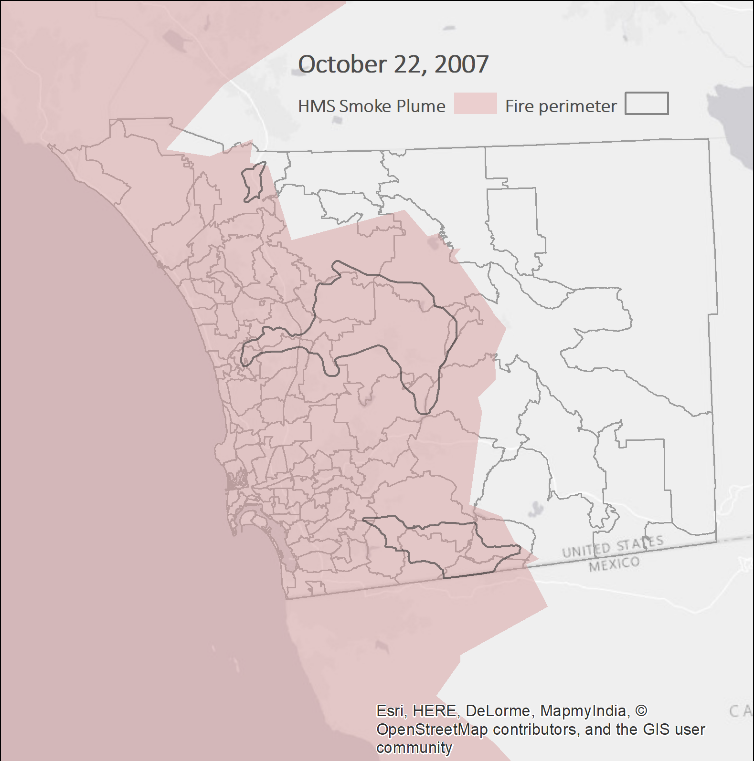 | 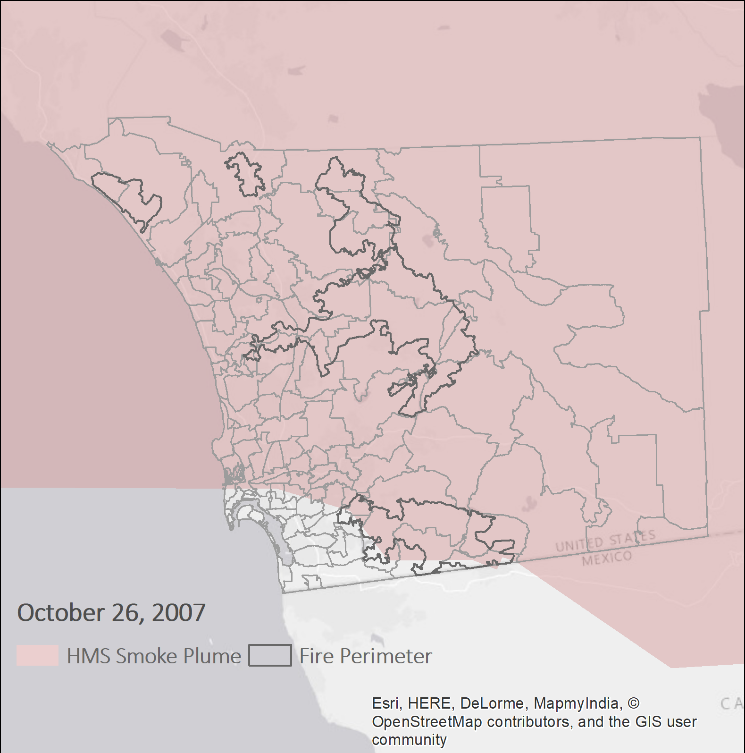 |
| --- | --- |
| 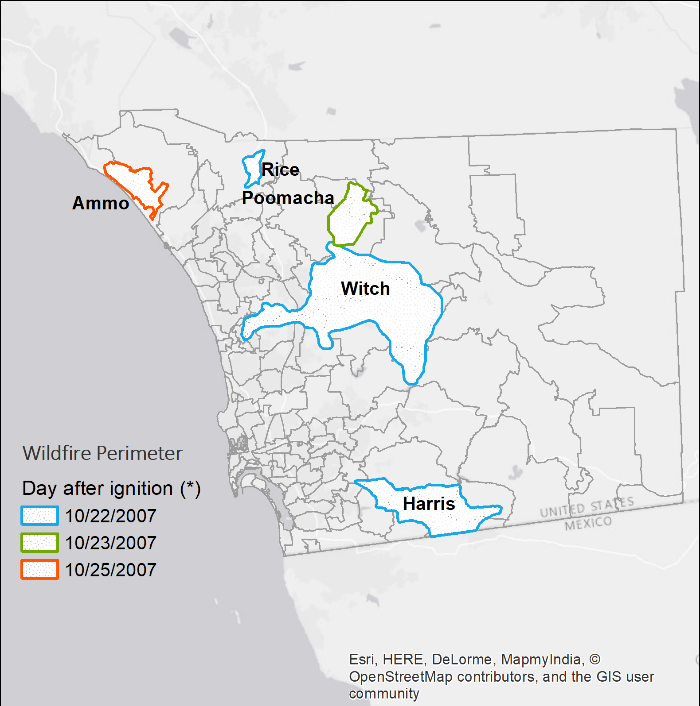 | 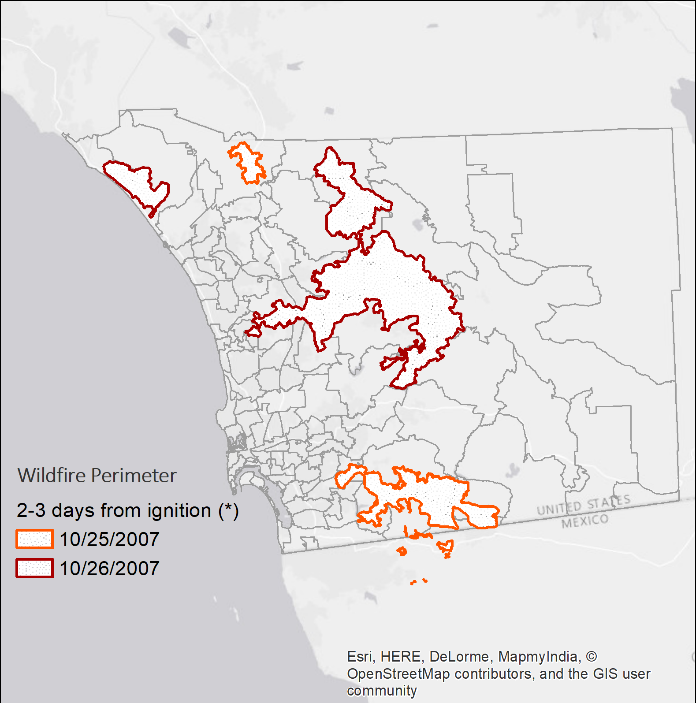 |

Figure S3: Counts of hospitalizations for individual zip codes on October 22 during the years 2004 (without wildfires; top) and 2007 (exposed to wildfire smoke; bottom). The latter recorded the highest number of hospitalizations in a single zip code (shown in dark red).


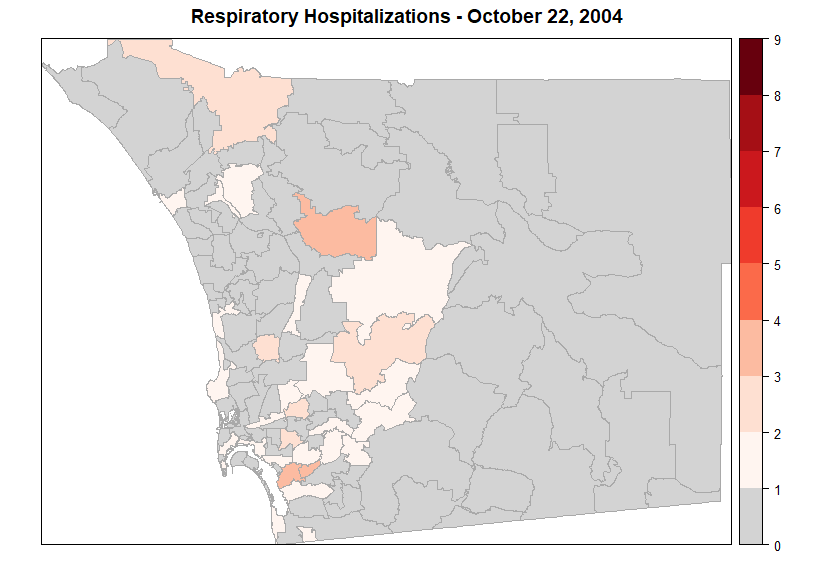


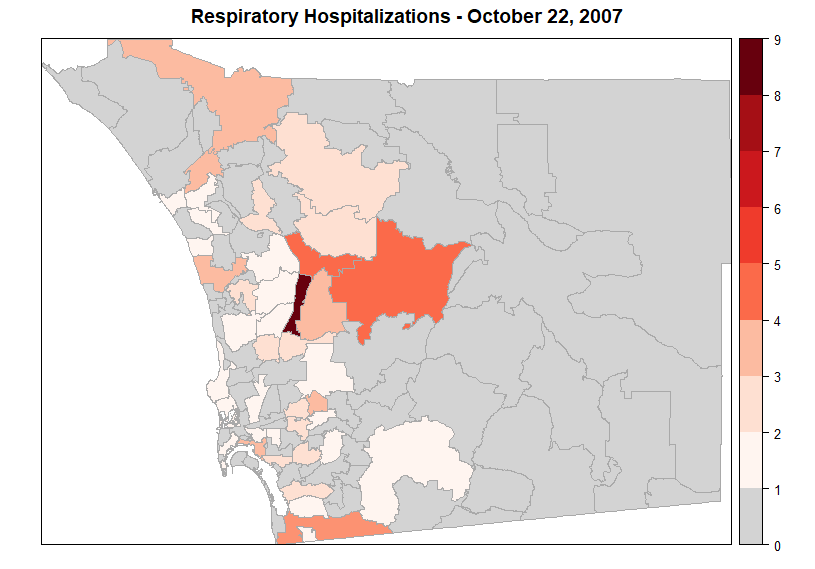


Figure S4: Interpolated surface of the posterior random spatial effects (mean values) resulting from the Bayesian Hierarchical Model (BHM). The mapped values represent mean excess hospitalizations for the main exposed period of October 22-26, 2007, with the highest values surrounding the Witch wildfire.


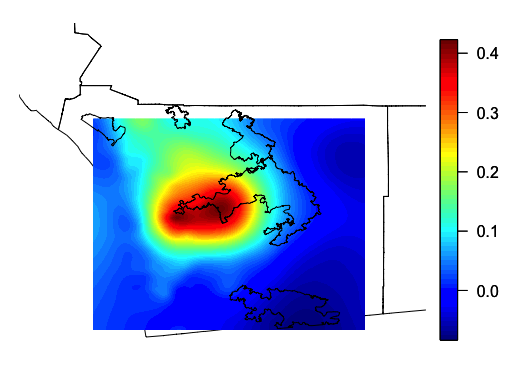


Figure S5: Anselin Local Moran’s I to examine spatial clusters of ZIP codes with high or low counts of excess hospitalizations as well as spatial outliers. Spatial outliers refer to ZIP codes with values of excess hospitalizations that are discrepant from the neighboring ZIP codes. For both years 2007 (top) and 2003 (bottom) , clusters of high counts of excess hospitalizations were found downwind of the largest wildfires.


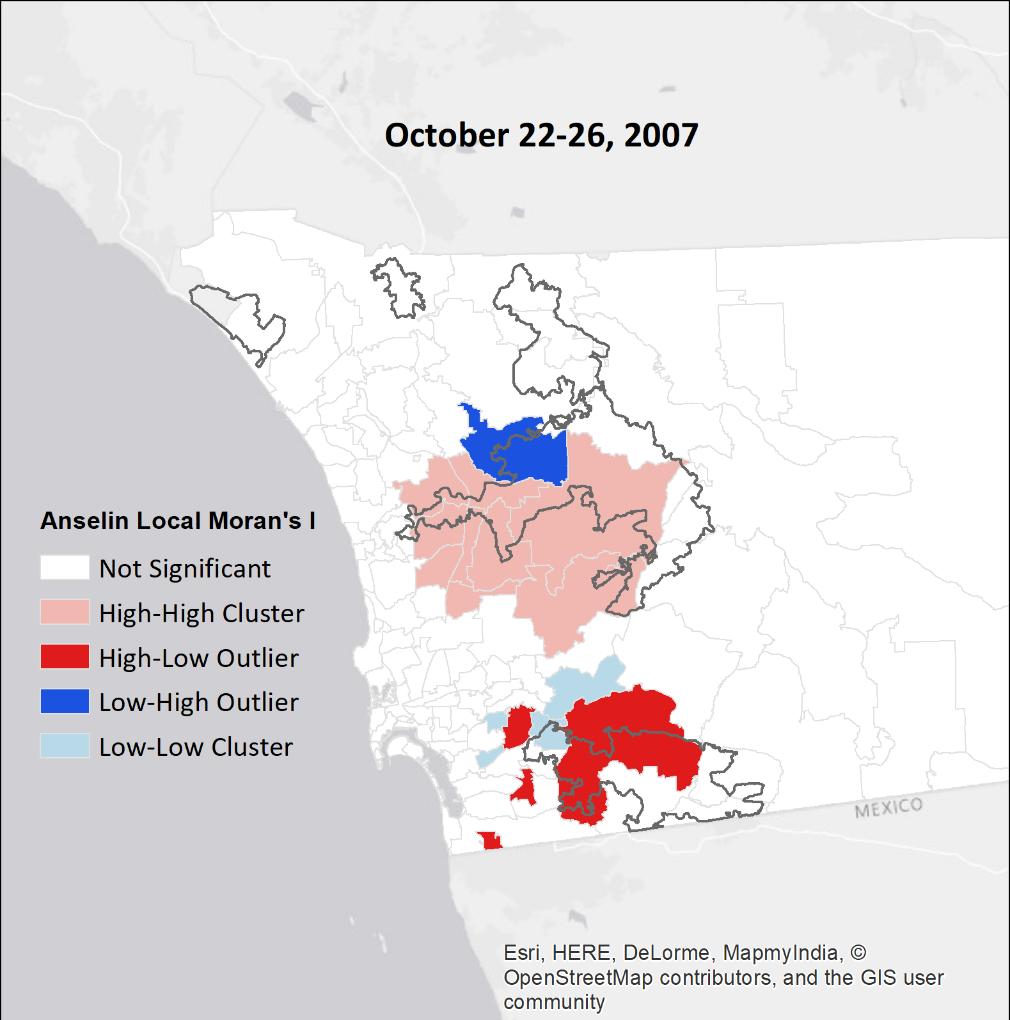

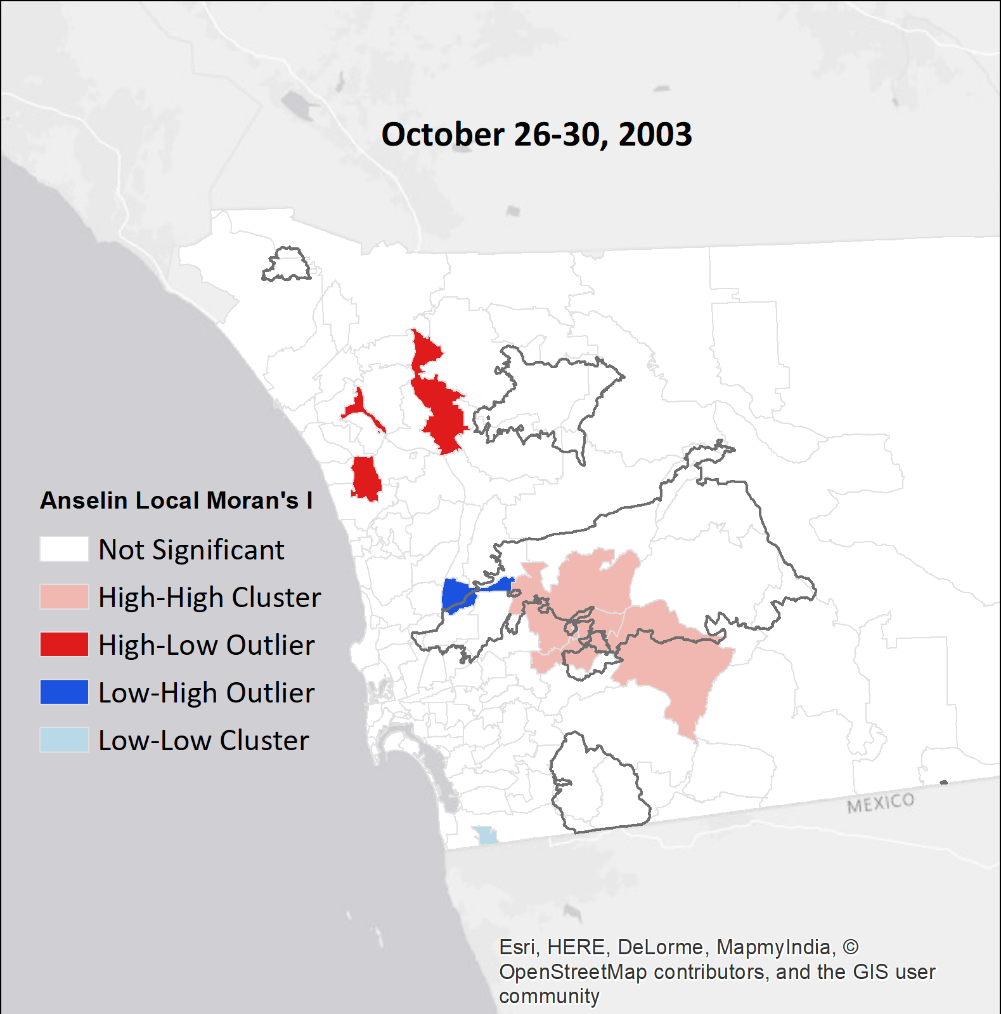


Table S1: For the Global Moran's I statistic, the null hypothesis states that the attribute being analyzed is randomly distributed among the features in the study area. The resulting not statistically significant p-values for years 2000 and 2004 show that the spatial processes promoting the observed values of excess counts in each ZIP code is random chance.

|  | **October 2000** | | **October 2004** | |
| --- | --- | --- | --- | --- |
|  | **22-26** | **26-30** | **22-26** | **26-30** |
| *Moran's I* | -0.0218 | -0.0008 | -0.0061 | -0.0315 |
| *z-score* | -0.5342 | 0.3694 | 0.1414 | -0.9450 |
| *p-value* | 0.5932 | 0.7118 | 0.8875 | 0.3447 |
|  |  |  |  |  |
